# Effective, Environmentally Friendly PVC Plasticizers Based on Succinic Acid

**DOI:** 10.3390/polym14071295

**Published:** 2022-03-23

**Authors:** Kerstin Ledniowska, Hanna Nosal-Kovalenko, Weronika Janik, Agata Krasuska, Dorota Stańczyk, Ewa Sabura, Maria Bartoszewicz, Aleksandra Rybak

**Affiliations:** 1 Łukasiewicz Research Network—Institute of Heavy Organic Synthesis “Blachownia”, Energetyków 9, 47-225 Kędzierzyn-Koźle, Poland; hanna.nosal@icso.lukasiewicz.gov.pl (H.N.-K.); weronika.janik@icso.lukasiewicz.gov.pl (W.J.); agata.krasuska@icso.lukasiewicz.gov.pl (A.K.); dorota.stanczyk@icso.lukasiewicz.gov.pl (D.S.); ewa.sabura@icso.lukasiewicz.gov.pl (E.S.); maria.bartoszewicz@icso.lukasiewicz.gov.pl (M.B.); 2 Department of Physical Chemistry and Technology of Polymers, PhD School, Silesian University of Technology, Akademicka 2a, 44-100 Gliwice, Poland; 3 Department of Physical Chemistry and Technology of Polymers, Faculty of Chemistry, Silesian University of Technology, Strzody 7, 44-100 Gliwice, Poland; aleksandra.rybak@polsl.pl

**Keywords:** plasticizer, succinate, phthalate, poly(vinyl chloride), renewable resources, migration resistance

## Abstract

The plasticizers used in this study were synthesized from renewable raw materials using succinic acid, oleic acid, and propylene glycol. Four environmentally friendly plasticizer samples were obtained; their chemical structures and compositions were confirmed by gas chromatography (GC) and infrared spectroscopy (FT–IR) analyses, and their physicochemical properties and thermal stability (TGA analysis) were investigated. The obtained ester mixtures were used as poly(vinyl chloride) (PVC) plasticizers and their plasticization efficiency was determined in comparison to traditional, commercially available phthalate plasticizers, such as DEHP (di(2-ethylhexyl phthalate) and DINP (diisononyl phthalate). Mechanical properties and migration resistance were determined for soft PVC with the use of three concentrations of plasticizers (40 PHR, 50 PHR, and 60 PHR). It was observed that the obtained plasticizers exhibited the same plasticization efficiency and were characterized with good mechanical and physical properties in comparison to commercial plasticizers. The tensile strength was approx. 19 MPa, while the elongation at break was approx. 250% for all tested plasticizers at a concentration of 50 PHR. Furthermore, plasticizer migration studies showed that the synthesized plasticizers had excellent resistance to plasticizer leaching. The best migration test result obtained was 70% lower than that for DEHP or DINP. The ester mixture that was found to be the most favorable plasticizer was characterized by good thermal and thermo-oxidative stability (5% weight loss temperature: 227.8 °C in air and 261.1 °C in nitrogen). The results of the research clearly indicate that the synthesized esters can provide a green alternative to toxic phthalate plasticizers.

## 1. Introduction

Plasticizers are an important class of compounds widely used as additives in the polymer industry to improve properties and polymer processing [1,2]. There are used in many polymers, but the flexible poly(vinyl chloride) (PVC) industry accounts for more than 90% of plasticizer sales by volume [3]. Flexible PVC is widely used in the production of a wide variety of materials, for example, cables, flooring, toys, medical equipment, and food packaging [4,5,6].

The number of currently produced commercial plasticizers covering such a wide range of PVC applications exceeds 500 [7,8]. Phthalate plasticizers (e.g., di(2-ethylhexyl) phthalate–DEHP) [4] are the largest group. Unfortunately, phthalates are compounds that migrate easily from the polymer matrix, have toxic effects on human health, and are hazardous to the environment [7,9]. Scientific studies have shown that phthalate plasticizers can increase the incidence of asthma, bronchitis, and cancer [10,11]. Therefore, the EU REACH Act prohibits the use of phthalic plasticizers in PVC products, such as children’s toys, food packaging or medical devices, which limits the growth of this type of plasticizer market [12,13]. The use of non-toxic and biodegradable alternatives to replace phthalates has become an inevitable trend [14,15].

The development of environmentally friendly polymer additives (bioplasticizers) is driving the plasticizers market due to the increasing demand for eco-friendly products and provides safety for human health and the environment [16]. The global bioplasticizer market was valued at USD 1364 million in 2021 and is expected to reach USD 1709 million by 2027. The Cumulative Annual Growth Rate (CARG) is expected to exceed 3.3% between 2021 and 2027 [17].

In recent years, several new plasticizers have appeared on the market, many of them based on renewable resources [18]. A typical example of a bioplasticizer that is commonly used in industrial practice is epoxidized soybean oil (ESBO). The raw material for its production is easily available, biodegradable, and has a relatively low price [19,20,21,22,23,24,25,26,27,28]. Another important group of bioplasticizers is esters [29], including citric acid esters [20,24,30,31], succinic acid esters [9,12,32,33,34,35,36], lactic acid esters [37,38,39], fatty acid esters [40,41,42], glycerol esters [43] or isosorbide esters [44,45,46].

Succinate esters are promising PVC environmentally friendly plasticizers that can replace toxic phthalate plasticizers. They are not only less toxic than phthalate plasticizers but also show better resistance to plasticizer migration from the polymer matrix. Elsiwi et al. [47] synthesized di-n-heptyl succinate from renewable feedstocks and obtained a biodegradable, effective, green PVC plasticizer. Similarly, promising results have been reported by Stuart et al. [34,35]. They investigated a series of bio-based succinate esters: diethyl- (DES), dibutyl- (DBS), dihexyl- (DHS), diethylhexyl- (DEHS), didecyl- (DDS), and didodecylsuccinate (DDoDS) as effective PVC plasticizers. It was shown that with the increase of the length of the alkoxy chains, the effectiveness of the succinate plasticizer increased until reaching the saturation point and this was equally or even more effective in PVC plasticization than DEHP. Additionally, succinates with shorter alkoxy chains (DES and DBS) can act as an effective plasticizer at higher concentrations. Erythropel et al. [9] also investigated the effect of the alkyl chain length of succinate molecules on plasticizing properties and biodegradation. The studies included changing the length of the side chains from n-ethyl to n-octyl. DHS was found to be the best PVC plasticizer, which is a compromise between longer molecules favorable for plasticization and shorter molecules favorable for biodegradation.

On the other hand, Erythropel et al. [32] obtained alternative compounds to DEHP based on 2-ethylhexyl diesters of maleic acid (cis isomer), fumaric acid (trans isomer), and succinic acid (saturated analog). They investigated the influence of molecular geometry on the biodegradation and plasticizing properties of PVC. It appeared that esters made from succinic acid or maleic acid would be suitable plasticizers for PVC as their plasticizing properties were comparable to DEHP, but it was found that the best green plasticizers were succinate esters of straight chain alcohol.

Polyesters based on succinic acid have been investigated by Gao et al. [48]. They synthesized four polyesters based on succinic acid and different diols such as 1,6-hexanediol, 1,4-butanediol, 1,3-propanediol, and ethylene glycol to obtain poly(hexane succinate) (PHS), poly(butylene succinate) (PBS), poly(propylene glycol succinate) (PPS), and poly(glycol succinate) (PGS). Only PHS showed good plasticizing properties on PVC with good migration resistance. Moreover, they observed that as the alkyl chain length of dihydric alcohols increased, the flexibility of plasticized PVC improved.

In several reports, succinic acid derivatives were used not as single plasticizers but in plasticizer mixtures [12,33,35] since most PVC compounds use plasticizer mixtures to optimize cost and physical properties. Erythropel et al. [12] investigated the effectiveness of plasticizers of succinate and maleate diesters mixed with PVC. Succinate and maleate diester plasticizers with linear side chains of four to eight carbons in length were as effective in plasticizing PVC as commercially available DEHP and DINCH, and in some tests, even the mechanical properties were improved. In turn, Chaudhary et al. [33] studied a mixture of DEHS with ESBO as a bio-based plasticizer for PVC. The study showed that the mixture of these two plasticizers minimized the disadvantages associated with using succinate alone as a plasticizer (improved migration) and also showed greater plasticization efficiency compared to ESBO alone. Stuart et al. [35] also investigated mixtures of succinate diesters as PVC plasticizers. DDS and DDoDS were mixed with DES, DHS, and DEHS. It was found that mixtures of these higher-molecular-weight succinate diesters and the lower-molecular-weight diesters were more effective in plasticizing PVC compared to single succinate plasticizers.

**Figure 1:**
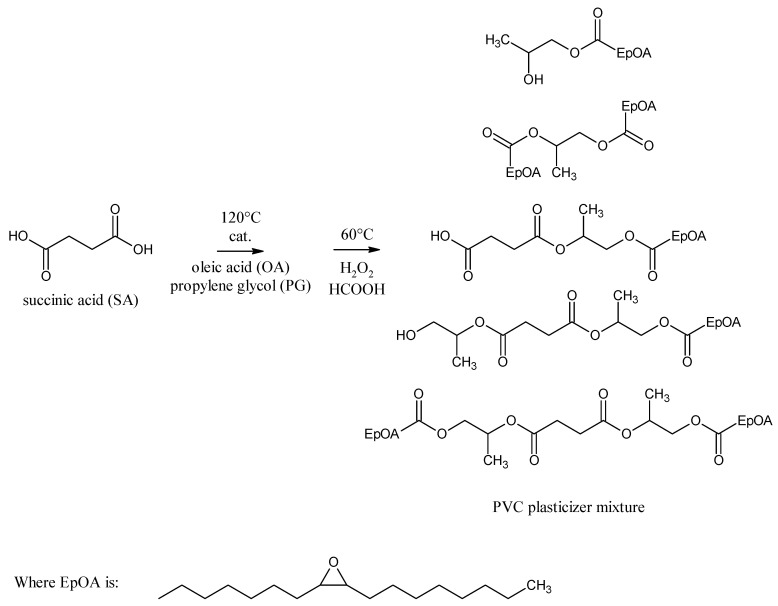
The chemical structure of the main compounds of the proposed mixture of epoxidized esters of succinic acid, propylene glycol, and oleic acid as a PVC plasticizer.

The aim of this work was to compose environmentally friendly raw materials and synthesis conditions to directly obtain as the esterification product a mixture of esters that would be an effective combination of PVC plasticizers. In this way, it will be possible to produce an effective plasticizer mixture in an one-pot production process while reducing production costs and energy consumption. In this study, we synthesized epoxidized mixed esters based on succinic acid, propylene glycol, and oleic acid, which can be used as environmentally friendly PVC non-migrating plasticizers. The use of a mixture of different ester structures as plasticizers can have a beneficial effect on their performance properties, especially when these esters differ significantly in hydrocarbon chain length. The chemical structure of the main components of the obtained plasticizer mixture is shown in Figure 1.

For the obtained plasticizer samples, GC, FT–IR, and TGA analyses were performed and the physicochemical properties were determined. The main goal of the work was to verify the compatibility and plasticization effectiveness of the obtained product with PVC. For this purpose, PVC samples plasticized with a mixture of epoxidized esters were prepared. Moreover, the plasticizing effect of the obtained plasticizer mixtures was compared with traditional phthalate plasticizers such as DEHP and DINP (diisononyl phthalate). In addition, the effect of plasticizer type on the functional properties, such as mechanical properties, hardness, density, and migration of soft PVC samples was investigated.

## 2. Materials and Methods

### 2.1. Materials

Oleic acid tech. 90.0% (Alfa Aesar, Ward Hill, MA, USA), propylene glycol pure p.a. (Chempur, Piekary Śląskie, Poland), succinic acid ≥ 99.5% (Pol-Aura, Zabrze, Poland), toluene pure p.a. (Chempur, Piekary Śląskie, Poland), and methanesulfonic acid > 99.0% (TCI, Zwijndrecht, Belgium), were used in this study to synthesize mixed esters. Formic acid 85.0% (Chempur, Piekary Śląskie, Poland), hydrogen peroxide 30.0% (Chempur, Piekary Śląskie, Poland), and di-sodium hydrogen phosphate dihydrate pure p.a. (Chempur, Piekary Śląskie, Poland) were used for epoxidation reaction and purification of epoxidized mixed esters.

Suspension PVC with a K value of 70 and a bulk density of 0.495 g/cm^3^ (Polanvil S-70, Anwil S.A., Włocławek, Poland) was used in this study. Calcium carbonate filler (Extra 1, Piotrowice II, Tarnobrzeg, Poland) and Ca/Zn thermal stabilizer (Baeropan R8890KA/2, Baerlocher GmbH, Unterschleißheim, Germany) were used as additives. PVC blends were plasticized with commercially available plasticizers, i.e., DINP (Jayflex, ExxonMobil, Machelen, Belgium), DEHP (Oxoplast O30, Grupa Azoty ZAK S.A., Kędzierzyn-Koźle, Poland), and with plasticizers synthesized (Samples 1, 2, 3, and 4) in this work.

### 2.2. Plasticizers Characteristic

#### 2.2.1. Gas Chromatography

The GC/FID analysis was performed on a PerkinElmer Autosystem XL gas chromatograph equipped with a flame ionization detector (FID) and on-column injector. A ZB-5HT capillary chromatography column was used (length = 15 m, internal diameter = 0.32 mm, film thickness = 0.10 µm) and it followed the temperature-programmed mode from 50 to 370 °C. The temperature of the injector was 50 °C, and the temperature of the detector was 380 °C. Helium was used as the carrier gas under a constant pressure of 70 kPa. The concentrations of particular groups of compounds (i.e., quantitative analysis) were established by an internal standard method.

For GC/MS analysis, a 7890A gas chromatograph (Agilent Technologies) was used, equipped with an MSD type 7000 GC/MS Triple Quad mass detector, computer station with Mass Hunter software, and a capillary column: DB-5HT (l = 30 m, internal diameter = 0.25 mm) with the temperature-programmed mode from 80 to 340 °C. The qualitative identification of the reaction mixture components was made on the basis of the analysis of their available standards (GC/FID), as well as the interpretation of the mass spectra recorded during the analysis (GC/MS), which were compared with the standard MS spectra from the NIST MS Search 2.0 computer library.

#### 2.2.2. Infrared Fourier Transformation (ATR–FTIR) Analysis

The infrared spectra were recorded on an FTIR Nicolet Model 6700 spectrophotometer with Omnic software from THERMO Scientific using the SMART ATR reflection attachment and ZnSe 60 crystals. FTIR tests were carried out based on the PN-ISO 6286:1994 standard [49].

#### 2.2.3. Thermogravimetric Analysis (TGA)

Thermal and thermo-oxidative stability were investigated using a Mettler Toledo TGA 2 system. Analytical samples were heated in an open platinum crucible (Pt 70 μL without lid) in the temperature range of 30–600 °C with a heating rate of 10 °C/min in a dynamic (100 mL/min) nitrogen or air atmosphere. Thermograms were analyzed using STAR Thermal Analysis software (version 15.0).

#### 2.2.4. Physicochemical Characteristic

The acid value, oxirane value, hydroxyl value, iodine value, and saponification value were determined according to Standard PN-EN 14104:2004, PN-C-89085-13:1987, DIN 53240-1:2013, PN-87/C-04281:1987, and PN-EN ISO 3657:2004 [50,51,52,53,54], respectively. The ester value was calculated from the equation where the acid value was subtracted from the saponification value. The water content was determined according to the Standard PN-ISO 760:2001 [55], performed on a 870 KF Titrino Plus titration assembly (Metrohm). The kinematic viscosity and density measurements were performed in accordance with Standard PN-EN ISO 3104:2004 and PN-EN ISO 3675:2004 [56,57], respectively. The tests were carried out at 20 °C, 40 °C, and 60 °C.

### 2.3. Soft PVC Characteristic

#### 2.3.1. Shore A Hardness

The Shore A hardness was determined according to the PN-ISO 868:2003 standard [58] using a Zwick hardness tester. Measurements were taken at five different locations of the test sample, and the average value was taken as the final result.

#### 2.3.2. Density

The density was determined according to the PN-EN ISO 1183-1:2019 standard [59] using a Mettler Toledo density determination kit AG-204. Measurements were performed in triplicate, and the average value was taken as the final result.

#### 2.3.3. Tensile Strength and Elongation at Break

Tensile strength and elongation at break were performed according to the PN-EN ISO 527-1:2020 and PN-EN ISO 527-2:2012 standards [60,61] using an Instron 4466 testing machine. Measurements were performed in five replications, and the average value was taken as the final result.

#### 2.3.4. Plasticizer Migration

Plasticizer migration was determined according to the PN-EN ISO 177:2003 standard [62]. Polyethylene discs were used as an absorbing material, and the migration was defined after seven days. Three replicates were performed for each sample, and the average value was taken as the final result.

### 2.4. Synthesis of Plasticizers–Epoxidized Mixed Esters of Succinic Acid, Propylene Glycol, and Oleic Acid (Sample 1–4)

Epoxidized mixed esters of succinic acid, propylene glycol, and oleic acid (Sample 1–4) were synthesized in three steps. The reactions were carried out in a 500 or 1000 mL four-necked glass reactor equipped with a variable speed stirrer, thermometer, nitrogen inlet tube, Dean-Stark trap, and condenser.

In the first step, propylene glycol (246.6 g) was esterified with oleic acid (653.5 g) using methanesulfonic acid (1.80 g) as a catalyst at 120 °C with vigorous stirring (400 rpm) and nitrogen bubbling. The water that was released as a by-product was removed from the reaction mixture azeotropically with the use of toluene (90.0 g) and collected in a Dean–Stark trap. The progress of the synthesis was monitored by sampling the reaction mixture every 60 min and determining the acid value of the reaction mixture. The process was carried out for 6 h until the acid value was less than 5 mg KOH/g, and then toluene was distilled from the reaction mixture on a vacuum evaporator.

In the next step, succinic acid (15.8–47.4 g) and a fresh portion of the catalyst, methanesulfonic acid (0.43–0.49 g), were added to the reaction mixture obtained in the first step of the synthesis (200.0 g). The water that was released as a by-product was removed from the reaction mixture azeotropically with the use of toluene (43.2–49.5 g) and collected in a Dean–Stark trap. The synthesis was carried out at 120 °C in the same way as in the first step. The progress of the synthesis was monitored by sampling the reaction mixture every 60 min and determining the acid value of the reaction mixture. The process was carried out for 4–6 h. In the second step, four different variants of the added succinic acid were used. Table 1 presents the tested variants of the proportion of COOH groups (derived from succinic acid) to OH groups (derived from propylene glycol monooleate and unreacted propylene glycol) with the amounts of each reactant.

In the third step, the reaction mixture from the second step was cooled to 50 °C and 28.4–20.7 g of formic acid (85 wt.% aqueous solution) was added. Thereafter, 265.6–193.7 g hydrogen peroxide (30 wt.% aqueous solution) was added drop by drop for about one hour. The mixture was vigorously stirred; the synthesis time, including the dropwise addition, was 4 h, and the reaction temperature was 60 °C. At the end of the synthesis, the reaction mixture was poured into a separating funnel and separated into two phases. The bottom layer containing mainly hydrogen peroxide, formic acid, and water was removed and the upper layer containing mixed esters was purified sequentially with 0.1 mol/dm^3^ Na_2_HPO_4_ solution and distilled water in an amount corresponding to the amount of the sample to obtain a pH of the epoxidized mixed esters close to 7. Water from purified epoxidized mixed esters was distilled using a rotary evaporator to a water content of less than 0.2 wt.%.

### 2.5. Preparation of Soft PVC Samples

The softened PVC formulations were prepared in an internal mixer (HAAKE^TM^ PolyLab^TM^ QC, Thermo Fisher Scientific). Initially, PVC resin (100 PHR), Ca/Zn stabilizer (4.5 PHR) and a certain amount of plasticizer (40, 50 or 60 PHR) were loaded into the mixer at 85 °C and at 36 rpm. Then, the temperature was allowed to rise to 100 °C and calcium carbonate (10 PHR) was added to the blend. Mixing was continued, and the temperature was allowed to rise to 120 °C. After this, the mixture was unloaded and allowed to cool to less than 40 °C. The blended appearance was a free-flowing powder (so-called dry-blend). Next, the blend was loaded back into the mixer at 180 °C and at 36 rpm. The process (gelation) was carried out for 10 min. After this time, the soft PVC samples were pressed (hydraulic press, type LP-S-50, Labtech Engineering Co) at 186 °C for 7 min in order to obtain the specimens for further characterization. Table 2 summarizes the prepared formulations. In the first twelve formulations, the effects of the content and the synthesized plasticizer type were evaluated, while in the last six, the effects of the content and the commercially available plasticizer type were studied for comparison.

## 3. Results and Discussion

### 3.1. Synthesis of Plasticizers

**Figure 2:**
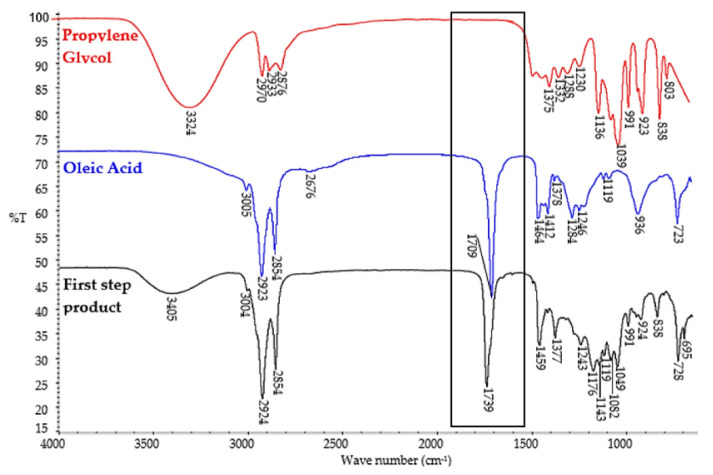
FT–IR spectra of raw materials (propylene glycol and oleic acid) and the first-step product.

The plasticizers were prepared in three steps. In the first step, esterification of propylene glycol with oleic acid was carried out to obtain as much propylene glycol monooleate as possible. This reaction is not selective and in addition to the expected propylene glycol monoesters, propylene glycol and oleic acid diesters were also formed. The obtained esters were analyzed by GC/MS and GC/FID (Table 3) as well as FT–IR (Figure 2).

The first confirmation of the esterification of propylene glycol with oleic acid was the shift of the C=O stretching vibration peak at 1709 cm^−1^ in oleic acid to 1739 cm^−1^ in the reaction product (Figure 2). The strong absorbance band at 1739 cm^−1^ is characteristic of C=O stretching vibration in esters. The formation of an ester bond in the products of the first synthesis step was also indicated by the presence of an absorbance band at 1176 cm^−1^ corresponding to a stretching C–O bond in the ester group. On the FT–IR spectra of the product of the first synthesis step, an absorbance band was also present at 3405 cm^−1^, corresponding to the stretching vibration of the OH group. This was expected since the main synthesis product is propylene glycol monooleate. However, the intensity of this band was significantly lower compared to the absorbance band corresponding to the stretching vibration of the OH group at 3324 cm^−1^ on the FT–IR spectra of propylene glycol. The FT–IR spectra of both oleic acid and the first step product (Figure 2) showed absorption bands at 3005 cm^−1^ and at 3004 cm^−1^, respectively which correspond to C–H stretching and deformation vibrations in unsaturated –HC=CH– bonds. This result was anticipated since monounsaturated fatty acid was used for the synthesis. The chemical composition of the obtained products was then determined. Table 3 shows the chemical compositions of the analyzed esterification products after the first step of synthesis. The identified components in Table 3 are ordered by their retention times. When planning this research, it was taken into account that in the obtained esterification products, besides the expected monoesters, propylene glycol diesters will also be present in certain amounts. The reaction mixture after the first synthesis step contained 58.4 wt.% of propylene glycol monooleate, 25.0 wt.% of propylene glycol dioleate, and 10.4 wt.% of unreacted propylene glycol. Very small amounts (0.4 wt.%) of dipropylene glycol were also formed during the reaction. The components described as volatile medium-molecular-weight components and volatile high-molecular-weight components are most likely propylene glycol monoesters and diesters of fatty acids other than oleic acid, respectively. Their presence is due to the fact that oleic acid tech. 90.0% was used for the synthesis.

**Figure 3:**
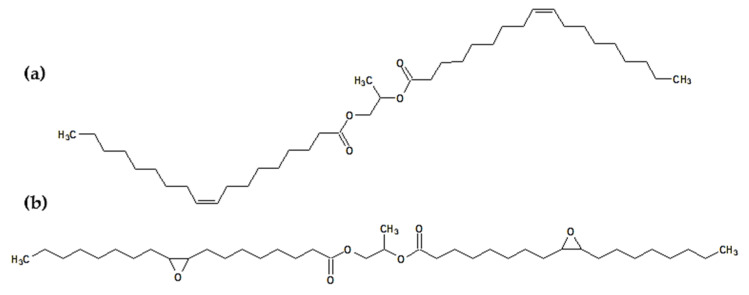
The chemical structure of (**a**) propylene glycol dioleate and (**b**) epoxidized propylene glycol dioleate.

The presence of propylene glycol dioleate may be an additional advantage since these compounds have a symmetrical linear structure with a long hydrocarbon chain on each side (Figure 3).

Figure 3 also shows the molecule of the epoxidized diester. This is due to the fact that the last step of the discussed synthesis was the epoxidation step. This means that in the final product, propylene glycol dioleate will also contain oxirane rings in its structure. Both structures (Figure 3) can act as effective plasticizers and will be important components of the final plasticizer mixture.

For the second step of the synthesis, the reaction mixture obtained in the first step was used. The aim of this step was to obtain the highest possible yield of succinic acid, propylene glycol, and oleic acid esters. For this purpose, four samples of the plasticizer, differing in the ratio of COOH groups (derived from succinic acid) to OH groups (derived from propylene glycol and propylene glycol monoester) were synthesized. The amount of succinic acid was calculated according to the mentioned proportion and was based on the data presented in Table 3. The esterification reaction was carried out in the same manner as in the first step. The reaction mixture was analyzed by GC/MS and GC/FID gas chromatography. Table 4 shows the chemical compositions of the analyzed esterification products after the second synthesis step.

The data in Table 4 indicate that almost all succinic acid reacted. Depending on the synthesis variant, its content in all products was very low: 0.0 wt.% (Sample 1), 0.1 wt.% (Sample 2), 0.2 wt.% (Sample 3), and 0.3 wt.% (Sample 4). However, the amount of succinic acid introduced into the reaction mixture clearly affected the chemical composition of the obtained products. The higher the amount of succinic acid used, the lower the content of unreacted propylene glycol in the reaction mixture. In Sample 1, where the least amount of succinic acid was used, the propylene glycol content in the product was 3.1 wt.%, while in Sample 4, where the highest amount of succinic acid was used, the propylene glycol content in the product was only 0.3 wt.%. The same correlation was observed for dipropylene glycol, which decreased to 0.1 wt.% in Sample 4. In addition, very small amounts of volatile low-molecular-weight components (0.5–0.1 wt.%) indicated that the obtained plasticizers contained small amounts of compounds that could easily migrate from the polymer matrix.

As expected, the second step reaction products contained esters of oleic acid and propylene glycol. However, it is worth nothing that the introduced amount of succinic acid affected not only the content of oleic acid monoesters but also diesters. In the variant in which the molar ratio of COOH groups to OH groups was 0.3: 1.0 (Sample 1), the content of propylene glycol monooleate was 38.2 wt.% and that of propylene glycol dioleate was 36.3 wt.%. On the other hand, in the variant in which the molar ratio of COOH groups to OH groups was 0.9:1.0 (Sample 4), the content of propylene glycol monooleate was 9.3 wt.% and that of propylene glycol dioleate was 26.2 wt.%. On this basis, it may be concluded that under the applied synthesis conditions, propylene glycol monooleate undergoes an esterification reaction with succinic acid, but it may also be assumed that propylene glycol mono- and dioleate undergoes a transesterification reaction with succinic acid. This was confirmed by the high content of free oleic acid, e.g., 8.8 wt.% in Sample 4 or 4.3 wt.% in Sample 3.

The main purpose of this synthesis step was to obtain succinic acid esters. According to the data in Table 4, the total content of succinic acid, propylene glycol, and oleic acid mixed esters was 11.6 wt.% (Sample 1), 12.0 wt.% (Sample 2), 15.2 wt.% (Sample 3), and 14.0 wt.% (Sample 4). At first glance, these appears to be relatively low values. However, it may be assumed, that under the applied synthesis conditions, succinic acid and propylene glycol may undergo an oligomerisation reaction resulting in oligoesters of propylene glycol and succinic acid in the reaction products. This was indicated by the high content of non-volatile components in the obtained products. The higher the amount of succinic acid used in the synthesis, the higher the content of non-volatile components: 2.0 wt.% (Sample 1), 22.9 wt.% (Sample 2), 18.0 wt.% (Sample 3), and 36.0 wt.% (Sample 4). In addition to non-volatile compounds, the plasticizer mixture also contained volatile medium- and high-molecular-weight components. The content of volatile medium- and high-molecular-weight components ranged from 2.3 wt.% to 1.1 wt.%, and they occurred near the retention time of propylene glycol monooleate and are most likely monoesters of fatty acids other than oleic acid. Their content was lower in response to more succinic acid used for synthesis. In turn, the volatile high-molecular-weight components listed in Table 4 are the components near the retention time of the propylene glycol dioleate and succinic acid, propylene glycol, and oleic acid mixed ester region. In this case, we suspect that these are analogous esters of fatty acids other than oleic acid.

These components have similar properties to the corresponding oleic acid esters. Their presence in certain amounts should not significantly influence the properties of obtained mixtures as PVC plasticizers.

The third step of synthesis was the epoxidation reaction, which was performed for each plasticizer sample in the same way, oxidizing the unsaturated bonds derived from oleic acid with performic acid. The third step was carried out to introduce oxirane groups into the plasticizer structure. The composition of the mixture after the third stage did not change; only unsaturated bonds were oxidized, which was confirmed by the determination of the oxirane value. The products obtained in the particular variants differed significantly in chemical composition, but all of their components have the potential to act as PVC plasticizers. In the next part of this work, it will be verified if their composition is an effective plasticizer.

**Figure 4:**
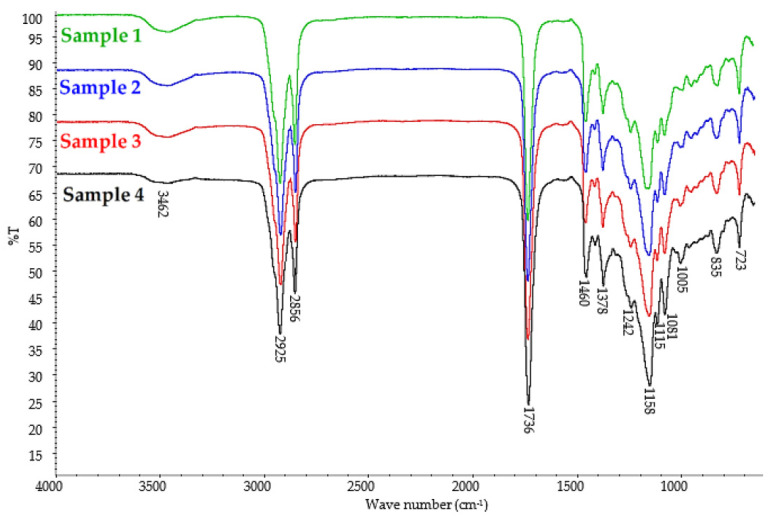
FT–IR spectra of epoxidized mixed esters (Samples 1–4).

The structures of the obtained esters (Samples 1–4) were confirmed by FT–IR spectroscopy (Figure 4).

The FT–IR spectra for all obtained plasticizers (Sample 1, 2, 3, and 4) were very similar, which is understandable since the individual samples differed only in the amount of succinic acid added in the second step of the synthesis. On the FT–IR spectra of Samples 1–4, at 1736 cm^−1^, there was a strong absorbance band corresponding to the stretching vibration of the double carbonyl bond (C=O) in the ester group, at 1158 cm^−1^, there was a strong absorbance band indicating a stretching C–O bond in the ester group, and at 1081 cm^−1^, a stretching C–O–CH2– bond occurred in the ester group. This confirms that the esterification reaction occurred in all reaction routes. The presence of a stretching C–O bond corresponding to the oxirane group at 835 cm^−1^ and the absence of deformation vibrations in the unsaturated –HC=CH– bond indicated that the epoxidation reaction was successful for all synthesized samples. The FT–IR spectra also showed weak O–H stretching vibration bonds of the alcohol group (Figure 4, Samples 1, 2, 3, and 4). The presence of this band corresponds to the hydroxyl groups in the unreacted diol used for the synthesis of the esters, as well as from the obtained monoesters. The presence of free propylene glycol and propylene glycol monoesters in the analyzed samples was also confirmed by GC analysis.

At 2925 cm^−1^ and 2856 cm^−1^, C–H stretching vibrations bands were present, and at 1460 cm^−1^, 1378 cm^−1^, and 723 cm^−1^, C–H deformation vibrations bands appeared, indicating hydrocarbon chains. The small band at 1115 cm^−1^ may be attributed to stretching vibrations in ethers because propylene glycol can undergo oligomerization reactions under esterification conditions, which was also confirmed by GC analysis (Table 4).

### 3.2. Properties of Epoxidized Mixed Esters

#### 3.2.1. Physicochemical Characteristics

The chemical properties of the synthesized epoxidized mixed esters of succinic acid, propylene glycol, and oleic acid are shown in Table 5.

All synthesized plasticizers were characterized by a low iodine value in the range of 1.1–2.8 g I_2_/100 g, which indicates the low content of unsaturated bonds in individual samples. This is related to the formation of oxirane rings in place of unsaturated bonds in the oleic acid moiety. The presence of oxirane rings in the synthesized plasticizers structure was also confirmed by the oxirane value (0.15–0.26 mol/100 g). It was noticed that as the amount of succinic acid added increased, the value of oxirane decreased. When the smallest amount of succinic acid was used for the synthesis of plasticizers (Sample 1), the oxirane value was 0.26 mol/100 g, while when the largest amount of succinic acid was used (Sample 4), the result was 0.15 mol/100 g. This is due to a decrease in the epoxidized oleic acid moiety and an increase in the succinic acid moiety in the mixture of the produced esters. An inverse relationship was observed for the acid value results–as the addition of succinic acid increased, the acid value also increased. Correspondingly, for Sample 1, it was 2.9 mg KOH/g, and for Sample 4, it was 29.4 mg KOH/g. Under synthesis conditions, succinic acid undergoes not only an esterification reaction but also a transesterification reaction (oleic acid substitution) as a result of which the acid number of the product is higher with higher amounts of succinic acid used. Hydroxyl values of 103.0 mg KOH/g, 94.0 mg KOH/g, and 95.4 mg KOH/g were obtained for Samples 1, 2, and 3, respectively, while Sample 4 had a much lower value of 59.9 mg KOH/g, which may indicate a lower unreacted hydroxyl group content and a higher conversion of oleic acid monoesters and propylene glycol. Therefore, they should have a higher ester value. This is in accordance with the results obtained from the ester value analysis (Table 5). Sample 4 was characterized by the highest ester value of 268 mg KOH/g. For the other samples, the ester values were lower: 251 mg KOH/g (Sample 3), 243 mg KOH/g (Sample 2), 218 mg KOH/g (Sample 1). All synthesized plasticizer samples had a low water content, less than 0.15 wt.%.

**Figure 5:**
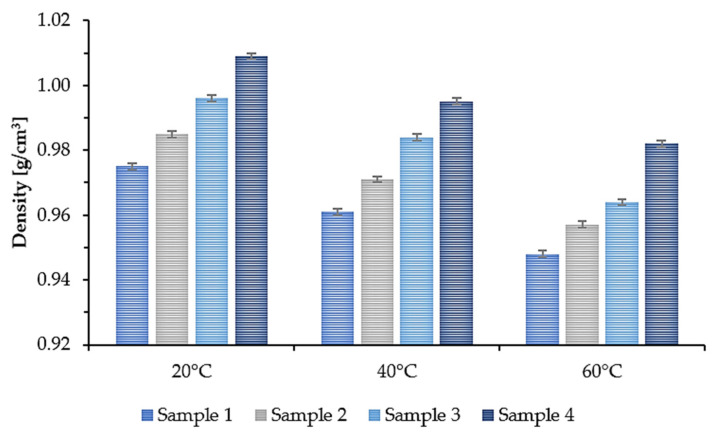
Density of epoxidized mixed esters (Samples 1–4) at 20, 40, and 60 °C.

**Figure 6:**
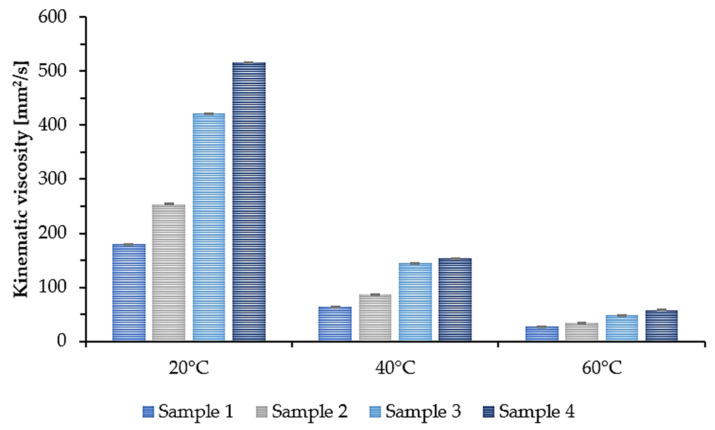
Kinematic viscosity of epoxidized mixed esters (Samples 1–4) at 20, 40, and 60 °C.

In many applications, the viscosity and density parameters of the plasticizer are of great importance. Therefore, the density and kinematic viscosity at 20 °C, 40 °C, and 60 °C were determined for the synthesized epoxidized plasticizers. The results are presented in the form of a graph. Figure 5 shows the density. Figure 6 shows the kinematic viscosity of the four plasticizers tested at three different temperatures.

As the temperature increased, the density and viscosity decreased. Additionally, a tendency can be noticed that for Sample 1, where the least amount of succinic acid was used, the density and kinematic viscosity values for each of the tested temperatures were the lowest. As the amount of added succinic acid increased, the density and kinematic viscosity values increased accordingly. At room temperature (20 °C), Sample 1 had a density of 0.975 g/cm^3^ and a kinematic viscosity of 179.3 mm^2^/s, while Sample 4 had a density of 1.009 g/cm^3^ and a kinematic viscosity of 516.4 mm^2^/s. This means that by changing the molar ratio of COOH groups derived from succinic acid to OH groups derived from propylene glycol and propylene glycol monoester, the viscosity of the synthesized plasticizers can be modified.

#### 3.2.2. Thermogravimetric Analysis (TGA)

**Figure 7:**
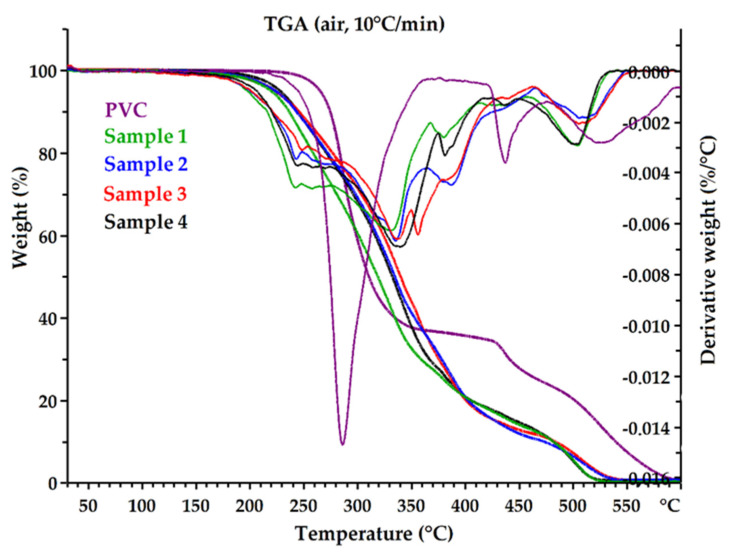
The TG and DTG curves of epoxidized mixed esters (Samples 1–4) and pure PVC in air.

**Figure 8:**
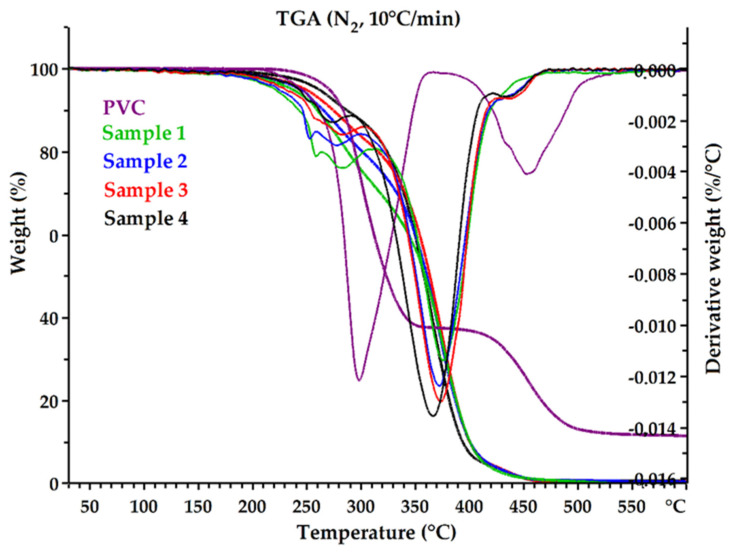
The TG and DTG curves of epoxidized mixed esters (Samples 1–4) and pure PVC in nitrogen.

One of the significant factors that determines the functional properties of epoxidized mixed esters is their thermal and thermo-oxidative stability. The thermal degradation of the received plasticizers and pure PVC was investigated in nitrogen (inert atmosphere) and in air (oxidative atmosphere). TG and DTG curves are shown in Figure 7 and Figure 8. Thermal and thermo-oxidative stability parameters, such as 5% weight loss temperature (T_d_^5%^), 10% weight loss temperature (T_d_^10%^), 90% weight loss temperature (T_d_^90%^), and DTG peak temperature (T_peak_) are shown in Table 6. All curves exhibited a multistage thermal degradation process with two distinct stages of weight loss and showed similar thermal behaviors (Samples 1 and 4). However, the initial degradation (weight loss 5% and 10%) clearly differentiated the samples. Sample 1 was found to have the lowest thermal stability compared to the other plasticizers, and Sample 4 had the highest stability. The parameters T_d_^5%^ and T_d_^10%^ increased from Sample 1 to Sample 4, in both nitrogen and air atmospheres. It follows that Sample 4 contained the lowest amount of low-molecular-weight constituents. Moreover, Sample 4 rapidly decomposed completely at high temperatures without generating a residue (T_d_^90%^ parameter was the lowest). It can be assumed that this was related to the amount of non-volatile components in each sample (Table 4). Sample 4 had the highest content of high-molecular-weight oligo esters, which are characterized by high thermal stability and low volatility.

Plasticizers are often low-molecular-weight compounds with lower thermal stability than PVC (e.g., DEHP) [63], which was also observed in this study. The temperature of 5% weight loss in the air for Sample 4 was 227.8 °C, while for pure PVC it was 264.9 °C, while in nitrogen it was 261.1 °C and 270.5 °C, respectively; the difference was not significant. The decomposition temperatures for the plasticizers themselves were significantly higher than the processing temperature of PVC, so they can be successfully used to produce flexible PVC.

### 3.3. Properties of Soft PVC Samples

#### 3.3.1. Physical Characteristics

On a macroscopic scale, all soft PVC samples were homogeneous, and the prepared samples were slightly yellowish in color changing to darker shades up to orange at a lower plasticizer content. According to the results shown in Table 7, all soft PVC samples had a similar density of about 1.3 g/cm^3^ and a hardness of Shore A approx. 95 for samples with 40 PHR of plasticizer, Shore A approx. 88 for samples with 50 PHR of plasticizer, and Shore A approx. 80 for samples with 60 PHR of plasticizer. PVC hardness depends on the plasticizer concentration: the higher the plasticizer efficiency, the lower the plasticizer concentration required. Since the hardness results were found to be similar for different plasticizers, it can be concluded that the plasticizers evaluated in this study showed the same efficiency as the commercially available plasticizers.

#### 3.3.2. Tensile Strength and Elongation at Break

**Figure 9:**
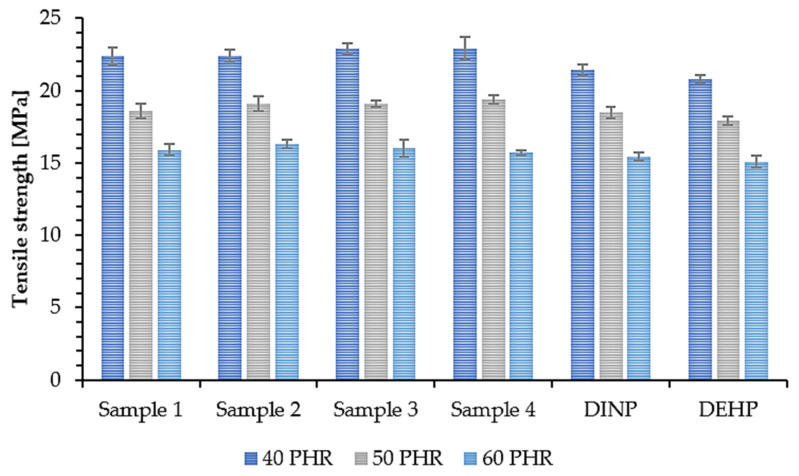
Tensile strength of soft PVC samples.

**Figure 10:**
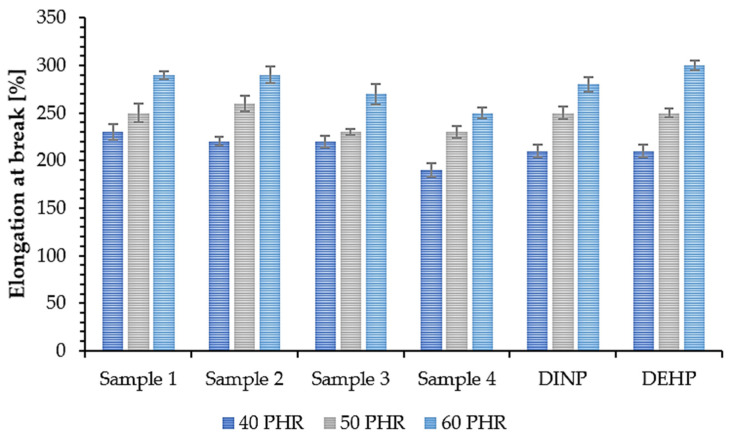
Elongation at break of soft PVC samples.

The mechanical properties of PVC samples plasticized with synthesized and commercially available plasticizers were determined to investigate the effect of plasticizer addition on their overall strength. The tensile strength (TS) of a material depends on the resin and additives used in its composition, i.e., the plasticizers [64]. Therefore, this property along with elongation at break (EB) is a good way to evaluate the efficiency of plasticizers. The obtained results of TS and EB are shown in Figure 9 and Figure 10. Samples 1, 2, 3, and 4 had the highest TS values (approx. 23 MPa (40 PHR), 19 MPa (50 PHR), and 16 MPa (60PHR)) with no significant differences between them. Samples prepared with commercial plasticizers, i.e., DINP and DEHP, had slightly lower TS values (about 21 MPa (40 PHR) and almost no difference for higher plasticizer contents, i.e., approx. 18 MPa (50 PHR) and approx. 15 MPa (60 PHR). This shows that the synthesized plasticizers had the same efficiency as commercially available plasticizers and in some cases, were even slightly better. Based on the results shown in Figure 10, it was observed that all samples had an EB of approx. 215% (40 PHR), 250% (50 PHR), and 280% (60 PHR). No significant differences were observed between soft PVC samples plasticized with synthesized and commercial plasticizers. These similarities in the mechanical properties of the samples are due to the chemical properties of the plasticizers used in this study; their interactions with the polymer were similar and therefore their effectiveness was also similar [65]. Relating the results of EB to the TS results, it can be seen that an increase in plasticizer concentration resulted in an increase in EB but a decrease in TS. A similar situation was observed by Lindström et al. [66], who reported the same relationship for samples plasticized with different contents of DEHP. Better mechanical properties of soft PVC samples plasticized with branched poly(butylene adipate) were also reported due to their higher efficiency [66]. This in turn was associated with a lower packing density of polymer chains. A higher degree of branching generates more mobile chain-ends and thus increases the free volume in the material and improves the mechanical properties of the sample [67].

#### 3.3.3. Plasticizer Migration

**Figure 11:**
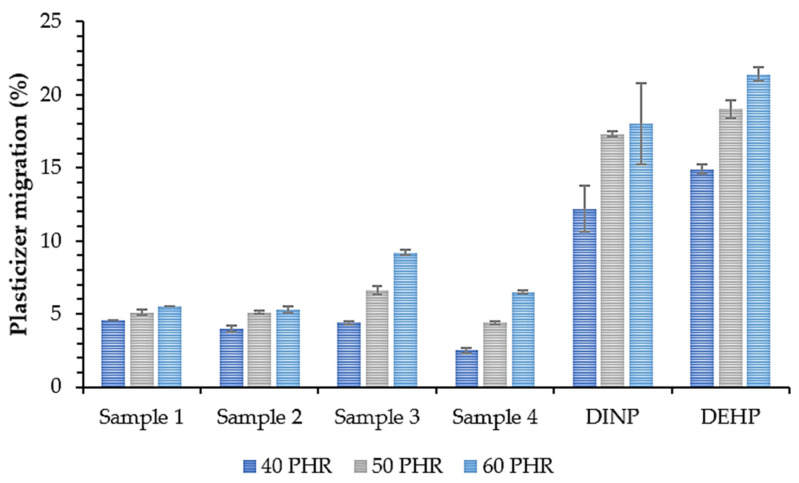
Plasticizer migration from soft PVC samples.

The effectiveness of a plasticizer depends on the molecular weight of a plasticizer and the ratio of individual components in a molecule, i.e., polar groups, non-polar polarizable groups, and non-polar non-polarizable groups [68]. The balance between the polar and non-polar parts of a molecule is also critical for controlling its compatibility [69]. The diffusion rate of the plasticizer in the polymer matrix is one of the most important factors determining its effectiveness. Some symptoms of plasticized sample instability are excessive and rapid sweating out of the plasticizer, leading to the deterioration of the mechanical properties [70]. Therefore, plasticizer migration tests were performed, and the results are shown in Figure 11. The migration of all synthesized plasticizers was significantly lower than that observed for the monomeric DINP and DEHP (approx. 70% lower migration for each plasticizer content). All produced esters were then characterized by superior migration resistance. The lowest migration values were noted for Sample 4, which contained plasticizers with fewer oxirane rings in the structure (oxirane value of 0.15 mol/100 g), but for its synthesis, the highest amount of succinic acid was used, which was practically completely incorporated into the structure of the obtained esters (0.3 wt.% unreacted succinic acid in Sample 4, Table 4). As a result, this product had the highest ester value (268 mg KOH/g). Soft PVC samples prepared with plasticizers with an oxirane value higher than or equal to 0.17 mol/100 g and an ester value lower than or equal to 251 mg KOH/g (Sample 3) had a slightly higher migration value than Sample 4. This low migration of Sample 4 may be due to its stronger interactions with the polymer matrix than the other plasticizers studied in this work. Van der Waals forces in the form of dipole–dipole interactions occur between the polar groups of the plasticizer such as carbonyl in ester bonds and chlorine atoms in the PVC matrix. Moreover, hydrogen bonds occur between the carbonyl groups of the plasticizer ester and the hydrogen attached to the carbon in the α position relative to the carbon–chlorine bonds in the PVC [66,71]. As suggested by the ester number, the concentration of ester groups in Sample 4 was the highest of all obtained plasticizers, and its interactions with the polymer were then also the strongest. Ester group interactions in the case of PVC plasticizers are therefore more important for limiting plasticizer migration than oxirane ring interactions.

The second factor influencing plasticizer migration is the molecular weight and volatility. GC and TG analyses clearly showed that the higher the amount of succinic acid introduced into the synthesis, the higher the amount of macromolecular non-volatile components in the obtained products. These components are probably oligomers of propylene glycol and succinic acid characterized by a high amount of ester groups. Larger molecules are more difficult to evaporate from the polymer matrix and therefore migrate less.

**Figure 12:**
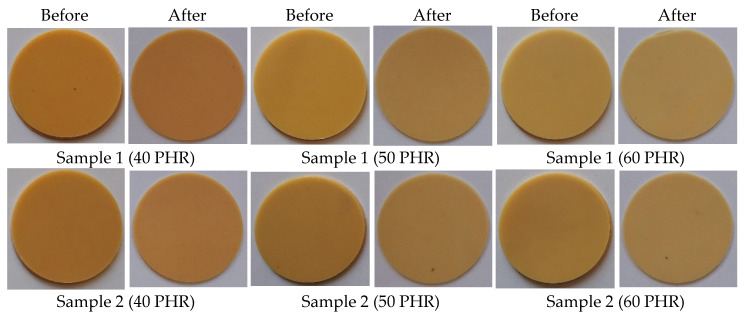

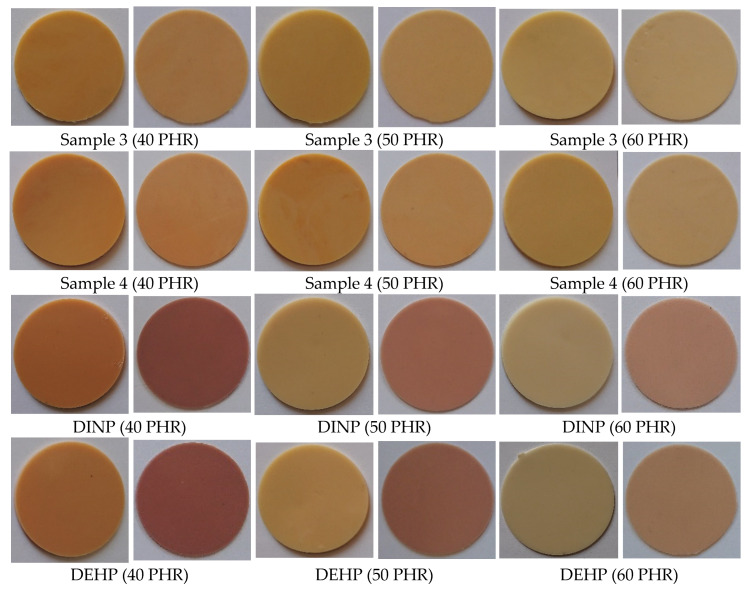
Soft PVC disc appearance–before and after migration.

The appearance and color of the material are some of the most important factors affecting product quality and customer attraction. As shown in Figure 12, samples plasticized with commercial plasticizers (DINP and DEHP) definitely changed their color after the migration test. Samples were yellow before the test and maroon after. For samples prepared with synthesized plasticizers, their color almost did not change at all. The color change of the material is related to the degradation of PVC. It is well known that PVC degrades by dehydrochlorination at elevated temperatures, resulting in conjugated double bonds in the polymer chains and a noticeable color change (from yellow to pink, orange, red, brown, and finally black) when the sequences of conjugated polyenes contain more than four to five double bonds [72,73,74,75,76]. In our study, the degradation was a result of a high migration of DINP and DEHP plasticizers shown in Figure 11, which makes the material more susceptible to degradation [77].

## 4. Conclusions

To avoid the negative effects caused by plasticizer migration and at the same time maintain the good properties of soft PVC, low-molecular-weight plasticizers were replaced with compatible and non-toxic ester plasticizers. In the present work, mixtures of different esters using succinic acid, oleic acid, and propylene glycol were synthesized by a three-step modification process and incorporated into PVC at 40, 50, and 60 PHR concentrations. The produced plasticizers were characterized by GC, TGA, FTIR, and physicochemical properties. Their plasticizing performance was mainly evaluated by the analysis of physicochemical properties, mechanical properties, and plasticizer migration of the soft PVC samples and was compared with those of the commercial plasticizers DINP and DEHP.

The synthesized plasticizers are a mixture of esters of succinic acid, propylene glycol, and oleic acid, which differ in the content of the respective esters. The higher the amount of succinic acid used in plasticizer synthesis, the higher the content of non-volatile compounds among the products obtained. This had a positive effect on their thermal stability and performance. Moreover, the physicochemical and mechanical properties of soft PVC samples suggested that in this study, plasticizers had the same efficiency as DINP and DEHP. Furthermore, plasticizer migration studies showed that the synthesized plasticizers had the best compatibility and plasticizing effects. Compared to DINP and DEHP, the migration was as much as 70% lower for each plasticizer concentration. Thus, due to their good compatibility, efficiency and thermal properties, the plasticizers synthesized in this research have the potential to replace petroleum-based plasticizers in PVC applications. Moreover, since they were produced from renewable, environmentally friendly, biodegradable, and readily available raw materials, the natural plasticizer mixtures will reduce the dependence on petroleum-based plasticizers. According to the obtained results, all evaluated samples exhibited adequate soft PVC properties.

## Figures and Tables

**Table 1:** Quantities of reagents in the second step of mixed ester synthesis.

Component	Sample 1	Sample 2	Sample 3	Sample 4
Molar ratio of COOH groups: OH groups (mol)	0.3: 1.0	0.5: 1.0	0.7: 1.0	0.9: 1.0
First step reaction mixture (g)	200.0	200.0	200.0	200.0
Succinic Acid (g)	15.8	26.3	36.8	47.4
Toluene (g)	43.2	45.3	47.4	49.5
Catalyst (g)	0.43	0.45	0.47	0.49

**Table 2:** Composition of soft PVC samples.

PVC Sample No.	Plasticizer Type	Plasticizer Amount	
		(PHR)	(g)
P1	Sample 1	40	12.8
P2	Sample 1	50	15.0
P3	Sample 1	60	17.1
P4	Sample 2	40	12.8
P5	Sample 2	50	15.0
P6	Sample 2	60	17.1
P7	Sample 3	40	12.8
P8	Sample 3	50	15.0
P9	Sample 3	60	17.1
P10	Sample 4	40	12.8
P11	Sample 4	50	15.0
P12	Sample 4	60	17.1
P13	DINP	40	12.8
P14	DINP	50	15.0
P15	DINP	60	17.1
P16	DEHP	40	12.8
P17	DEHP	50	15.0
P18	DEHP	60	17.1

**Table 3:** Compositions after the first step of plasticizer synthesis by GC/MS and GC/FID.

Composition (wt.%)	First Step Product
Propylene glycol	10.4
Dipropylene glycol	0.4
Volatile low-molecular-weight components	0.5
Oleic Acid	0.7
Propylene glycol monooleate	58.4
Volatile medium-molecular-weight components	2.4
Propylene glycol dioleate	25.0
Volatile high-molecular-weight components	2.2

**Table 4:** Composition after the second step of plasticizer synthesis based on GC/MS and GC/FID.

Composition (wt.%)	Sample 1	Sample 2	Sample 3	Sample 4
Propylene glycol	3.1	2.1	0.6	0.3
Dipropylene glycol	0.4	0.3	0.1	0.1
Succinic Acid	0.0	0.1	0.2	0.3
Volatile low-molecular-weight components	0.3	0.5	0.2	0.1
Propylene glycol succinate	3.5	4.1	2.5	1.9
Oleic Acid	1.5	5.7	4.3	8.8
Propylene glycol monooleate	38.2	22.1	21.4	9.3
Volatile medium-molecular-weight components	2.3	2.0	1.6	1.1
Propylene glycol dioleate	36.3	27.3	34.6	26.2
Succinic acid, propylene glycol, and oleic acid mixed esters	11.6	12.0	15.2	14.8
Volatile high-molecular-weight components	0.8	0.9	1.4	1.1
Non-volatile components	2.0	22.9	18.0	36.0

**Table 5:** Chemical properties of epoxidized mixed esters (Samples 1–4).

Properties	Sample 1	Sample 2	Sample 3	Sample 4
Acid value (mg KOH/g)	2.9	6.4	14.4	29.4
Oxirane value (mol/100 g)	0.26	0.21	0.17	0.15
Hydroxyl value (mg KOH/g)	103.0	94.0	95.4	59.9
Iodine value (g I_2_/100 g)	2.8	1.9	1.1	2.0
Saponification value (mg KOH/g)	221	249	265	297
Ester value (mg KOH/g)	218	243	251	268
Water content (wt.%)	0.15	0.09	0.10	0.09

**Table 6:** Thermal and thermo-oxidative stability parameters of epoxidized mixed esters (Samples 1–4) and pure PVC.

Sample	T_d_^5%^ (°C)	T_d_^10%^ (°C)	T_d_^90%^ (°C)	T_peak_ (°C)
air				
Pure PVC	264.9	274.1	539.3	285.7
Sample 1	219.6	236.1	481.5	330.7
Sample 2	223.6	242.9	472.3	335.3
Sample 3	223.1	245.3	483.2	336.5
Sample 4	227.8	244.0	481.0	338.8
nitrogen				
Pure PVC	270.5	283.0	-	297.8
Sample 1	236.6	259.5	400.1	400.1
Sample 2	243.7	265.0	400.2	400.2
Sample 3	248.8	274.1	399.9	399.9
Sample 4	261.1	286.6	393.5	393.5

**Table 7:** Physical characteristic of the soft PVC samples.

PVC Sample No.	Shore A Hardness	Density (g/cm^3^)
P1	93.8 ± 0.4	1.315 ± 0.002
P2	87.4 ± 0.5	1.283 ± 0.002
P3	78.2 ± 0.4	1.249 ± 0.002
P4	95.2 ± 0.4	1.322 ± 0.001
P5	86.6 ± 0.5	1.286 ± 0.002
P6	80.2 ± 1.9	1.251 ± 0.012
P7	96.6 ± 0.5	1.326 ± 0.002
P8	91.4 ± 0.5	1.297 ± 0.001
P9	84.0 ± 0.7	1.267 ± 0.004
P10	97.4 ± 0.5	1.335 ± 0.002
P11	93.4 ± 0.5	1.305 ± 0.003
P12	85.0 ± 1.2	1.274 ± 0.003
P13	95.0 ± 0.7	1.310 ± 0.004
P14	86.8 ± 0.8	1.278 ± 0.002
P15	78.4 ± 0.5	1.248 ± 0.002
P16	93.0 ± 0.7	1.319 ± 0.002
P17	84.6 ± 0.5	1.289 ± 0.001
P18	74.6 ± 1.1	1.255 ± 0.004

## Data Availability

Not applicable.
